# Feasibility of 3D vascular reconstruction for preoperative planning in robotic living donor nephrectomy: a retrospective pilot study

**DOI:** 10.3389/fsurg.2026.1690717

**Published:** 2026-02-19

**Authors:** Mohamed El-Mahrouk, Beatrice Lukenaite, Todor Stoyanov, Antonia Geisler, Andri Lederer, Robert Karitnig, Robert Sucher, Saulius Mikalauskas

**Affiliations:** Division of General, Visceral and Transplantation Surgery, Department of Surgery, Medical University of Graz, Graz, Austria

**Keywords:** 3D reconstruction, live donor nephrectomy, robotic surgery, surgical planning, transplantation

## Abstract

**Introduction:**

Patient safety is crucial in donor nephrectomy. Individualized 3D reconstructions from CT imaging are gaining popularity for improving preoperative planning. This study evaluates whether such models enhance anatomical assessment and surgical preparation in robotic live donor nephrectomy.

**Methods:**

This retrospective pilot study included 24 patients who underwent robotic live donor nephrectomy between 2022 and 2025. 3D models were reconstructed by the surgeon using MED EINS software and compared to conventional CT reports regarding vascular anatomy.

**Results:**

Of the 24 nephrectomies, 75% were left-sided. No intraoperative complications occurred; minor postoperative complications (Clavien-Dindo I/II) were observed in 16.7% of cases. Donors had a mean age of 52.9 years and a mean BMI of 28.0. Most had a single renal artery (83.3%) and vein (91.7%). The mean operative time was 205 min, and hospital stay averaged 6.4 days. Donors were often parents or partners (each 41.7%). 3D reconstructions showed high concordance with CT reports. In one case, a second artery was missed on the CT report but visible on the 3D model, demonstrating its utility for detecting anatomical variants.

**Conclusion:**

3D reconstructions have the potential to support and refine preoperative planning in robotic donor nephrectomy by enhancing anatomical visualization. However, their clinical benefit should be further validated in prospective studies.

## Introduction

The only therapy for end-stage renal disease is kidney transplantation. However, demand significantly exceeds the available supply: in 2024 alone, more than 10,000 individuals in the Eurotransplant region were waiting for a kidney transplantation (1). Due to this organ shortage, living donation plays a particularly important role in reducing waiting times.

The kidney is an organ with frequent anatomical variations. A meta-analysis showed accessory renal arteries occur in about 21,1% (2). Therefore, it is of all importance that the surgeon carefully analyzes preoperative cross-sectional imaging—particularly computed tomography (CT)—to ensure the procedure is carried out as safe as possible. Nevertheless, small accessory renal arteries can sometimes be difficult to identify on CT imaging. This raises the question of how a surgeon can further improve preoperative planning to enhance donor safety. One method that is increasingly being adopted in clinical practice is individualized 3D reconstruction. Nowadays, various medical software tools are available that can reconstruct DICOM data from CT or MRI scans into 3D models. Several studies have demonstrated the clinical utility of three-dimensional reconstructions across a range of surgical specialties, including general and abdominal surgery, orthopedics, and neurosurgery (3–6). A major benefit of 3D reconstruction is its contribution to individualized preoperative planning, especially in technically demanding procedures such as complex liver resections and pulmonary segmentectomies (7, 8).

3D reconstruction has been successfully applied across various organ systems. Therefore, the aim of this study is to retrospectively evaluate its additional value in terms of visualization during robotic donor nephrectomies. Does this approach allow for improved detection of anatomical variations?

## Methods

This pilot study is a single-center retrospective analysis focusing on patients who underwent robotic donor nephrectomy between November 2022 and April 2025. In this period, 24 robotic donor nephrectomies were performed at our institution.

### Surgical technique

All live donor nephrectomies were performed using the Da Vinci Xi robotic system. Access was gained via a Pfannenstiel incision with placement of an Alexis wound protector and establishment of pneumoperitoneum. Three trocars were placed pararectally on either the right or left side. An additional assistant trocar was inserted at the umbilicus if required, depending on intraoperative findings. Vessel identification and division were performed using standard Hemo-lok clips (9).

### 3D reconstruction

**Figure 1:**
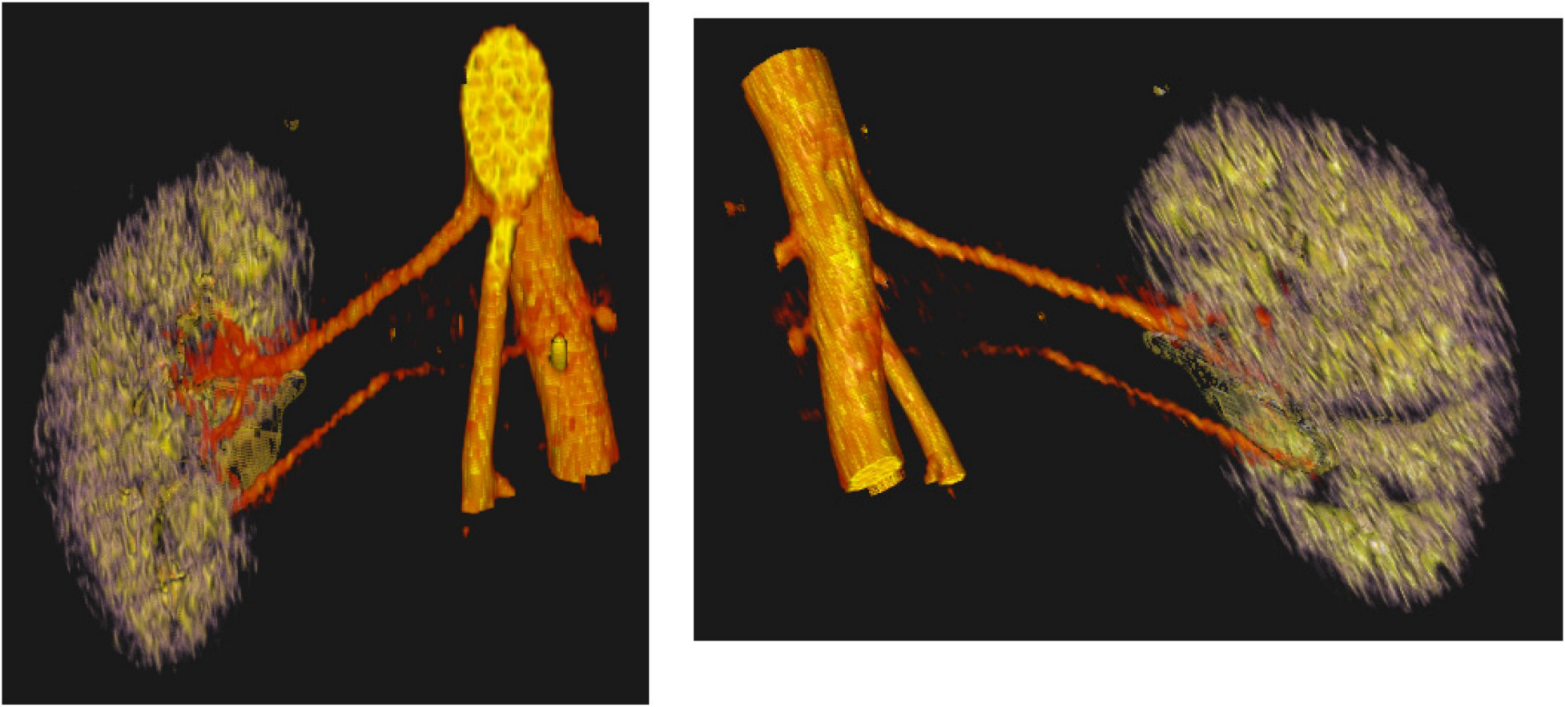
Patient A: ventral (left) and dorsal (right) views of the kidney showing a caudal renal artery that was not reported in the initial CT assessment.

**Figure 2:**
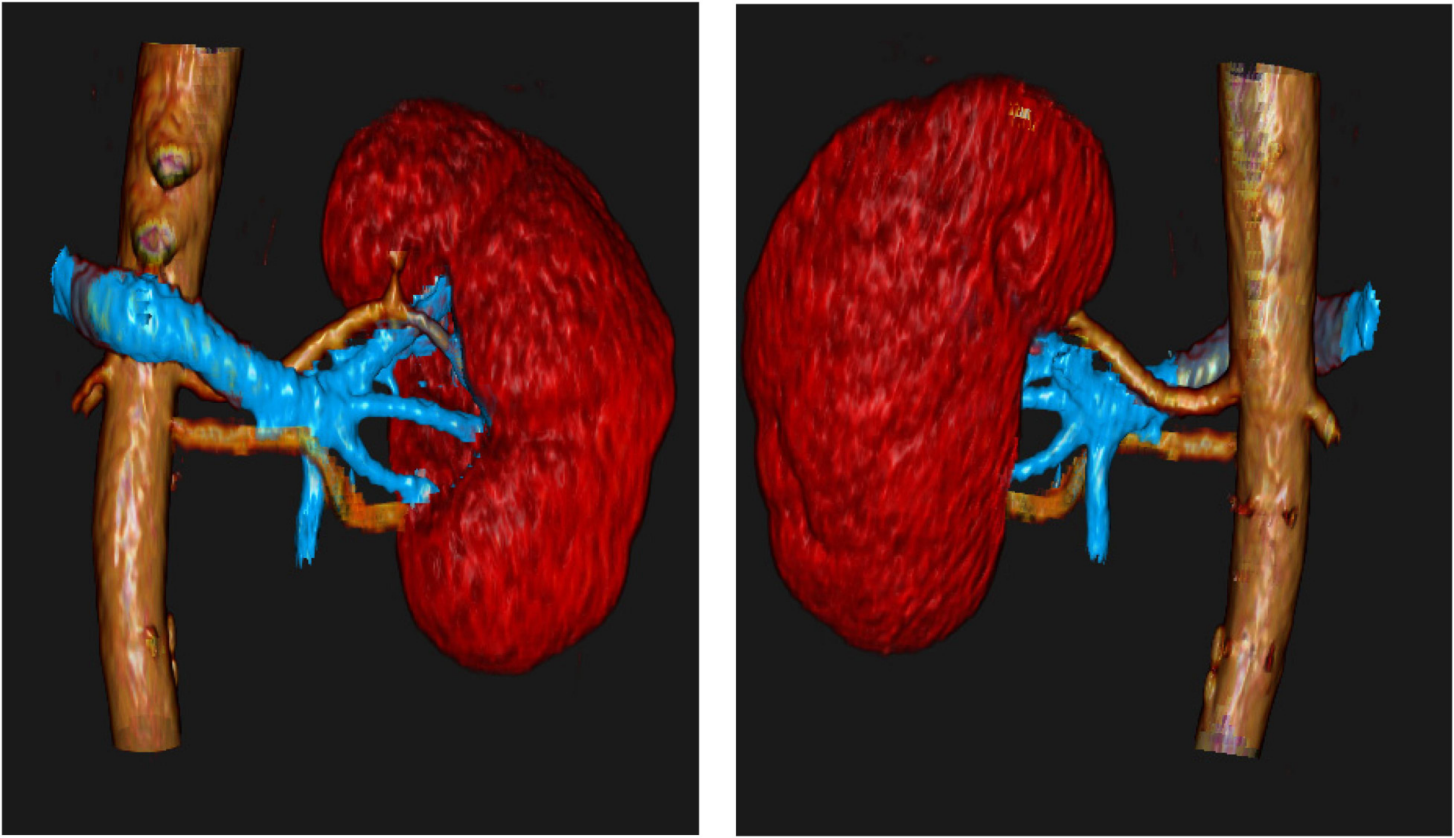
Patient B: ventral (left) and dorsal (right) views of the kidney demonstrating two renal arteries.

**Figure 3:**
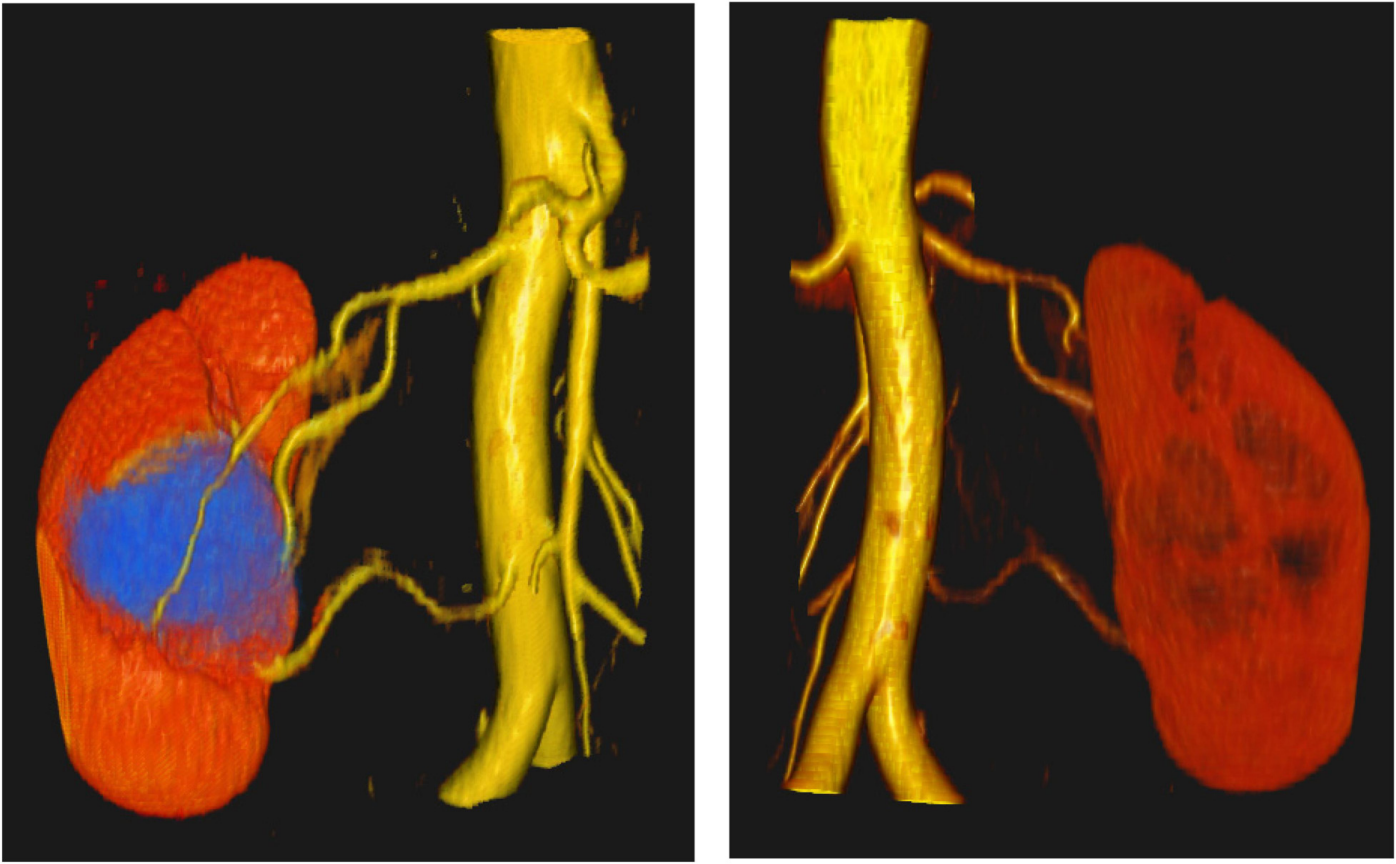
Patient C: ventral (left) and dorsal (right) views of the kidney showing two renal arteries. A renal hilum cyst is highlighted in blue in the left image.

The 3D reconstruction was performed by the surgeon using a product developed by the company “MED EINS—Medical Solutions.” The average time required to perform the reconstruction was approximately 30 min. The MED EINS system uses a certified stereoscopic 3D planning software to convert original DICOM imaging data (e.g., CT or MRI) into interactive holographic visualizations. Specific phases of a CT scan, such as the arterial phase, can be selectively used to better visualize certain structures—for example, arteries. In addition, the software offers various visualization options for the 3D models, including customizable color schemes and density settings for individual structures (Figures 1–3) (10). All CT scans of the 24 patients were reconstructed using the program und we analyzed if there were differences between the CT report and the 3D reconstruction.

### Statistic

Given the small sample size and the exploratory nature of this pilot/feasibility study, only descriptive statistics were planned to summarize the data, and no inferential statistical comparisons were intended or performed. Categorical variables were described using absolute and relative frequencies, while means and standard deviations were calculated for ordinal and continuous variables. For this purpose, the statistical software SPSS version 29 was used.

## Results

In our cohort (*n* = 24) 75% of the live donor nephrectomies were performed on the left side (*n* = 18), while 25% were on the right side (*n* = 6). Female donors comprised 70.8% of the cohort (*n* = 17), while males made up 29.2% (*n* = 7). Regarding the ASA classification, 33.3% of donors were classified as ASA 1 (*n* = 8), 58.3% as ASA 2 (*n* = 14), and 8.3% as ASA 3 (*n* = 2). The majority of donors were either parents (41.7%, *n* = 10) or partners (41.7%, *n* = 10) of the recipients, while friends (8.3%, *n* = 2), as well as cousins and siblings (each 4.2%, *n* = 1), accounted for the remainder. There were no intraoperative complications reported in any case (100%, *n* = 24). Postoperative outcomes were uneventful in the majority of cases, with 83.3% of donors (*n* = 20) experiencing no complications. Isolated complications occurred in single cases (4.2% each), including asymptomatic leg swelling, urinary tract infection, pneumonia, and polyuria.

For continuous variables, the mean age at surgery was 52.9 ± 10.4 years (range 27–67), mean weight was 81.2 ± 16.1 kg (range 55–109), mean height was 170.1 ± 8.5 cm (range 158–190), and mean BMI was 28.0 ± 4.9 (range 19.3–39.7). The average surgery length was 205.1 ± 39.0 min (range 152–310). For the subset of 20 donors with detailed operative data, console time averaged 155.1 ± 44.4 min (range 61–267), instruments active time averaged 129.7 ± 32.9 min (range 58–189), number of instruments used was 3.6 ± 1.1 (range 3–7), and instrument changes averaged 3.7 ± 4.2 (range 0–17). Hospital stay averaged 6.4 ± 1.4 days (range 4–9), with post-surgery hospital stay averaging 5.0 ± 1.2 days (range 3–7). Preoperative creatinine was 0.8 ± 0.2 mg/dL (range 0.5–1.2), increasing to 1.3 ± 0.3 mg/dL at discharge (range 0.8–1.9).

All 24 CT scans were retrospectively reconstructed in 3D, demonstrating a high degree of concordance with the original radiological reports.

In 83.3% of cases (*n* = 20), donors had a single renal artery and vein. Vascular anomalies were identified in four cases (16.7%): two patients (8.3%) had dual renal arteries and veins, while the other two (8.3%) had two arteries with a single vein. When examined separately, a single artery was present in 83.3% (*n* = 20) of cases, and two arteries in 16.7% (*n* = 4). Similarly, one vein was observed in 91.7% (*n* = 22), with two veins in 8.3% (*n* = 2). Notably, in one case, the CT scan reported only a single renal artery, but 3D reconstruction revealed a second artery that was confirmed intraoperatively during the operation.

## Discussion

Ensuring donor safety remains the foremost priority in living donor nephrectomy, as the procedure is performed on healthy individuals who must not be exposed to unnecessary risks. Consequently, the operating surgeon must conduct a meticulous evaluation of preoperative cross-sectional imaging to identify any anatomical anomalies in advance and avoid unexpected intraoperative findings that could increase morbidity and compromise outcomes. In recent years, growing interest has been directed toward the potential role of patient-specific 3D reconstructions in improving surgical planning. Several studies across different surgical specialties have demonstrated the advantages of 3D models in enhancing anatomical understanding, facilitating operative strategy, and potentially reducing complication rates (3–6).

While 3D reconstruction is not a novel technology *per se*, its specific application in the setting of donor nephrectomy has not been discussed in the literature. This lack of evidence served as the impetus for our study, in which we sought to explore its potential utility in this highly delicate surgical context. Using standard preoperative CT imaging, we generated individualized 3D reconstructions for all patients in our cohort. We observed a high degree of concordance between the 3D models and the corresponding radiological CT reports. Notably, in one patient, the 3D reconstruction revealed a previously unreported accessory renal artery (Figure 1). The caudal vessel, located near the aorta, exhibited poor contrast enhancement, which may have contributed to its omission in the initial radiological report. Nonetheless, the 3D model enabled clear and unambiguous identification of the additional artery, even in the absence of specialized radiological expertise. Even in cases where vascular variants were correctly identified on conventional imaging, the 3D reconstructions provided a more intuitive, spatially coherent, and readily interpretable representation of the anatomy—potentially facilitating better intraoperative orientation.

To demonstrate the clarity and clinical utility of the 3D reconstructions, two additional models are presented. Figure 2 clearly visualizes two renal arteries along with the renal vein, offering a comprehensive view of the vascular anatomy. Figure 3 further showcases the model's ability to depict not only parenchymal and vascular structures but also pathological findings, such as a renal hilum cyst highlighted in blue.

Nevertheless, the broader implementation of routine 3D reconstruction raises important economic and logistical considerations. In our cohort, all live donor nephrectomies were performed safely and without intraoperative or postoperative complications—even in the absence of 3D models during the actual surgery. A relevant limitation of our study is that all procedures were conducted by highly experienced transplant surgeons who may possess the expertise to identify and navigate anatomical variations without additional visualization tools. It remains an open question whether 3D reconstruction might offer even greater value for less experienced surgeons or in training settings, where enhanced anatomical insight could translate into measurable improvements in safety and performance.

To determine the true clinical benefit of 3D reconstruction in donor nephrectomy, prospective studies with larger sample sizes are required. Ideally, such investigations should be randomized and include objective outcome parameters such as operative time, complication rates, conversion to open surgery, and donor morbidity. Only through such rigorous evaluation can the role of 3D reconstruction in enhancing donor safety and surgical outcomes be conclusively established.

### Limitations

This retrospective pilot study is limited by its relatively small cohort of 24 patients, which restricts the generalizability of the findings. However, the sample size was considered sufficient to assess the feasibility of 3D vascular reconstruction in the setting of robotic living donor nephrectomy and to identify practical strengths and limitations of this approach. The quality of the 3D reconstructions remains highly dependent on the quality of the DICOM data derived from CT imaging. In addition, the conclusions are largely based on subjective assessments rather than objective clinical endpoints, which may limit their robustness. Consequently, the true clinical value and impact of 3D reconstruction should be confirmed in larger, prospective studies using standardized outcome measures and long-term follow-up.

## Conclusion

Robotic donor nephrectomy is a reliable and effective technique for kidney procurement, offering the advantages of minimally invasive surgery with favorable perioperative outcomes. Although left-sided nephrectomy remains preferred in most centers due to more favorable vascular anatomy and longer renal veins, findings from this retrospective pilot cohort indicate that right-sided robotic donor nephrectomy can also be performed safely.

Within this context, individualized 3D reconstruction represents a technically feasible adjunct for enhanced anatomical visualization and preoperative planning. Despite the limited cohort size, the number of cases included was sufficient to assess feasibility and to demonstrate high anatomical concordance with conventional imaging, including the identification of vascular variants that may be underreported on standard CT evaluation. By improving anatomical orientation, 3D reconstruction has the potential to refine surgical planning, reduce intraoperative uncertainty, and support patient safety.

Nevertheless, while feasibility has been demonstrated, the definitive clinical benefit of 3D reconstruction—such as improvements in operative efficiency, complication rates, or donor outcomes—remains to be established. Larger, prospective studies with standardized endpoints, ideally incorporating randomized designs, are required to determine its true clinical value and to define its role in routine preoperative assessment.

## Data Availability

The raw data supporting the conclusions of this article will be made available by the authors, without undue reservation.
